# Nicotine Pouch Patterns of Use in a 10-Week Prospective Study

**DOI:** 10.7759/cureus.100915

**Published:** 2026-01-06

**Authors:** Lindsay Reese, Elliott H McDowell, Brian Erkkila, Tryggve Ljung

**Affiliations:** 1 Life Sciences, Philip Morris International (PMI) R&amp;D, Philip Morris Products S.A., Neuchâtel, CHE; 2 Regulatory, Philip Morris International (PMI) US Corporate Services, Stamford, USA; 3 Scientific Engagement, Philip Morris International (PMI) US Corporate Services, Stamford, USA; 4 Oral Products, Swedish Match AB North Europe, Stockholm, SWE

**Keywords:** nicotine pouches, post-market surveillance, switching behavior, tobacco harm reduction, tobacco use behavior

## Abstract

Background: Nicotine pouch (NP) product use has increased in the US, but limited data are available on how NPs are used and if they affect the use of other tobacco and/or nicotine products (TNPs), specifically transition away from more harmful TNPs such as cigarettes.

Methods: This prospective, observational study gathered information on daily use patterns of combustible and non-combustible TNPs (cigarettes, cigars, pipe tobacco, hookah, e-cigarettes, oral smokeless tobacco (ST)) and reasons for use among current adult NP users (n=346, ≥18 years old during the 2017-2018 study period) recruited with canister stickers in the 11 states where *ZYN*™ (NP-Z) was first sold. All analyses performed were descriptive in nature; values are provided as percentages with 95% confidence intervals, means with standard deviations, or medians with ranges, as appropriate.

Results: The proportion of participants who used NP-Z and smoked cigarettes at least one day a week at baseline decreased from 15.9% (12.0-19.8%) to 8.1% (5.2-11.0%) over the study period. Nearly half of them stopped smoking by week 10 (8.1% (5.2-11.0%) to 4.9% (2.6-7.2%)). Among those who used NP-Z and moist snuff, use of the latter declined from 15.0% (11.2-18.8%) to 7.5% (4.7-10.3%). Overall, 24.0% (19.5-28.5%) of participants who used NP-Z and other TNPs at baseline reported exclusive NP-Z use by the end of the 10-week study.

Conclusion: Patterns of use among early NP-Z adopters indicate that NPs can be acceptable replacements for other TNPs, particularly cigarettes and oral ST.

## Introduction

Nicotine pouches (NPs) are a rapidly growing tobacco and/or nicotine product (TNP) category. Sales of NPs in the US increased more than sixfold between 2019 and 2022 [1]. These tobacco leaf-free products do not contain many of the harmful and potentially harmful constituents (HPHCs) found in traditional smokeless tobacco (ST) products or cigarette smoke [2,3]. Importantly, people who exclusively use NPs have markedly lower exposure to HPHCs compared to those who use combusted tobacco [4,5]. This relatively new type of TNP has potential application in tobacco harm reduction if it is used to displace more harmful TNPs [6,7], but there is a lack of published data on the real-world use of NPs.

ZYN™ (NP-Z; Swedish Match NA, Richmond, VA) was initially test-launched in 50 stores in Colorado in 2014. The regional launch in June 2016 covered 11 western states, and it was launched nationally in April 2019. NP-Z are available in two concentrations (3 and 6 mg) and 10 variants (five mint, three coffee, citrus, cinnamon, and two unflavored). These products were submitted under a premarket tobacco product application to the US Food and Drug Administration (FDA) on March 4, 2020. A marketing-granted order authorizing NP-Z sale was issued in January 2025 [8], after a review determined that permitting marketing of the new products was appropriate for the protection of public health [9].

This prospective, observational, actual use study aimed to describe real-world patterns of NP use among adult (legal age 18+ during the November 2017 to April 2018 study period, changed to 21+ on December 20, 2019) early adopters of NP-Z over a 10-week period. The secondary objectives were to explore TNP patterns of use among NP-Z users and to compare the tendencies of participants to use the product with other TNPs, in complete replacement of other TNPs, or to quit all TNPs.

## Materials and methods

Study design

A cross-sectional, web-based survey was conducted between November 2017 and April 2018 by Kantar Health (now Oracle Life Sciences; Austin, TX). The study comprised two parts. In the first, past 30-day data on TNP use and perceptions were collected retrospectively among NP-Z users and non-users. The recruitment process and the retrospective results were previously published [10]. In the second, participants provided prospective data on TNP use in an e-diary over a 10-week period (Supplementary Materials). This was a descriptive actual use study with no control group.

Ethical and regulatory requirements

Ethical approval was obtained from the Sterling Institutional Review Board (Atlanta, GA) before the study began, and all participants provided electronic consent before completing any assessments. The study was carried out in accordance with the US FDA Center for Tobacco Products (CTP) draft guidance on data for human studies designed to evaluate the risks and benefits to the population as a whole [11]. Since the time of the study, this guidance has been finalized [12]. The survey was administered by Kantar Health in accordance with the requirements of their Quality System, which conforms to the International Standard for Market Research (Certification Number: 1019).

Participants

The recruitment process was described previously [10]. In brief, participants were recruited through stickers placed on ~290,000 product canisters in ~4,500 retail stores across 11 western states from November 27 to December 11, 2017. A total of 1,266 NP-Z users completed a web-based retrospective study to provide a descriptive analysis of patterns of TNP use and perceptions of health risks [10]. They were then invited to participate in a 10-week prospective study. All participants received compensation for their time spent completing the surveys.

Inclusion and exclusion criteria

The inclusion criteria were (1) legal age for TNP use (18+ at the time of the study), (2) able to read and speak English, (3) resident of a state where NP-Z was sold (Arizona, California, Colorado, Idaho, Montana, Nevada, New Mexico, Oregon, Utah, Washington, Wyoming), (4) current NP-Z use every day or some days, and (5) provided electronic informed consent. The exclusion criteria were (1) responding “don’t know” or “decline to answer” to specific demographic questions; (2) employment as a physician or in the fields of market research, marketing, advertising, or TNP manufacture; and (3) taken part in another research study on tobacco in the previous two weeks.

Study assessments

Sociodemographics: Participants provided their age, sex, race/ethnicity, level of education attained, marital status, and household income in the past 12 months.

TNP use: Participants were asked to complete a daily five-minute survey that asked which TNPs had been used the previous day (12:00 AM to 11:59 PM) and the number of times each type of TNP was used, resulting in 70 daily surveys completed. Every 14 days, the daily survey was extended by five minutes to obtain additional information (intention to quit and reasons for NP-Z use), resulting in a total of five bi-weekly surveys completed. If a participant missed a daily survey, they received a reminder call. For inclusion in the final dataset, participants needed to complete a minimum of five of the seven daily e-diaries each week.

Average daily consumption (ADC) and weekly use were derived from the daily surveys. The ADC was the number of products consumed for cigarettes, cigars, NPs, snus, moist snuff (dips), and chewing tobacco (pinches) and the number of sessions for e-cigarettes, pipe tobacco, and hookah/water pipe. Participants who reported using a TNP every day for a given week based on non-missing daily surveys (e.g., usage for 5/5, 6/6, or 7/7 daily surveys) were considered “every day” users, while those who reported using a TNP at least one day, but not every day, based on non-missing daily surveys were considered “some day” users. Participants who did not report any TNP use based on non-missing daily surveys (e.g., usage for 0/5, 0/6, or 0/7 daily surveys) were considered “not at all” users. Derived outcomes based on the survey items were based on the approach employed in the U.S. Population Assessment of Tobacco and Health (PATH) study for observing current TNP use [13].

Weekly use of NP-Z with other TNPs during the 10-week observational period was derived from the daily reporting of TNP use and used to stratify participants into one of six groups: NP-Z only, NP-Z + cigarettes, NP-Z + oral ST (e.g., moist snuff, chewing tobacco, or snus), NP-Z + other TNP (excluding cigarettes and oral ST), NP-Z + all other (e.g., NP-Z + cigarettes + snus, NP-Z + cigarettes + snus, NP-Z + snus + cigars, etc.), and no NP-Z use.

Quitting was defined as recording no TNP use of any kind in weeks 9 and 10. Complete replacement was defined as dual use in week one, but recording only NP-Z use in weeks 9 and 10.

Intention to quit: Participants’ intention to quit each TNP was assessed using the Motivation to Stop Scale (MTSS) [14]. The MTSS consists of one item with seven response options ranging from one (lowest) to seven (highest level of motivation to stop), also including “Don’t know.” The MTSS has been validated for cigarettes [14] and was adapted here for other TNPs. Consistent with published research, the mean MTSS score is reported [15].

Reasons for NP-Z use: One item in the biweekly survey assessed why participants had used NP-Z, and they were allowed to select multiple options from 21 possible reasons.

Adverse events: Only unsolicited adverse events (AEs) or product complaints spontaneously reported by study participants or on their behalf were collected, which were all assessed by a healthcare professional. An AE included any event that started after participants provided informed consent through the end of the data collection period.

Data analysis

Data from participants who completed all 10 weeks were included. For cases where data were absent due to incomplete daily surveys, no values were imputed. Daily TNP use patterns of use outcomes (e.g., average daily use for each TNP) were calculated based on the number of non-missing entries. Overall, 40% of participants did not have missing entries, 44% missed at most one day a week, and 16% missed two days a week. On average, respondents completed 6.8 days per week. The analyses performed for this observational study were descriptive in nature. All data were compiled and analyzed in Statistical Product and Service Solutions (SPSS, version 23; IBM SPSS Statistics for Windows, Armonk, NY). Descriptive statistics were applied to understand the distributions of sociodemographic and outcome variables (using raw data, i.e., prior to any aggregation required for the final presentation of results). Numeric variables are reported as mean ± standard deviation (SD) or median (range) as appropriate. Categorical variables are reported as a number (percentage). Total sample size and number of missing observations are reported where applicable. Respondents with values for variables that were illogical or deemed unreliable, as determined by the underlying distribution, were considered for removal prior to performing the main analyses.

## Results

Participants

**Figure 1:**
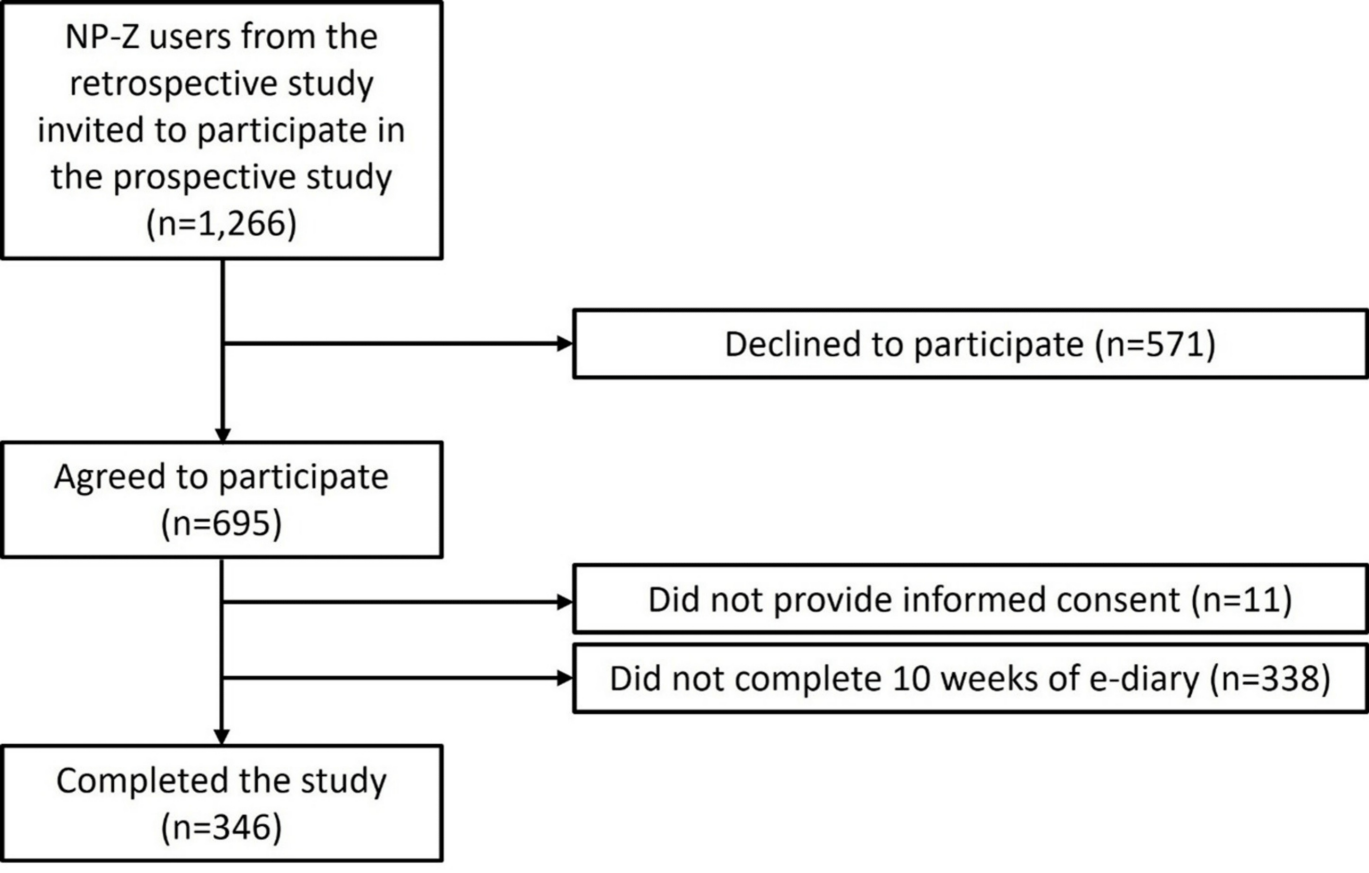
Study flow diagram

A total of 346 (27.3%) participants from the retrospective study [10] completed the 10-week prospective study (Figure 1). Table 1 lists their sociodemographic characteristics. Most were white (89.9%), male (88.2%), ≥25 years old (83.5%), and had attended college (80.3%). A majority (62.4%) reported annual household income ≥$50,000. Half (50.9%) were married.

**Table 1:** Participant (N=346) sociodemographic characteristics

Characteristic	n, %
State of residence	
Arizona	8 (2.3%)
California	53 (15.3%)
Colorado	39 (11.3%)
Idaho	42 (12.1%)
Montana	32 (9.2%)
Nevada	13 (3.8%)
New Mexico	18 (5.2%)
Oregon	66 (19.1%)
Utah	35 (10.1%)
Washington	38 (11.0%)
Wyoming	2 (0.6%)
Age (years)	
18–20	10 (2.9%)
21–24	47 (13.6%)
25–34	156 (45.1%)
35–44	90 (26.0%)
45–54	37 (10.7%)
≥55	6 (1.7%)
Sex	
Male	305 (88.2%)
Female	41 (11.8%)
Race/ethnicity	
White	311 (89.9%)
Black	1 (0.3%)
Hispanic	14 (4.0%)
Asian or Pacific Islander	1 (0.3%)
Native American	3 (0.9%)
Mixed racial background	13 (3.8%)
Other	3 (0.9%)
Highest education level	
Less than high school	2 (0.6%)
Some high school, no diploma	6 (1.7%)
High school diploma/equivalent	60 (17.3%)
Some college, no degree	140 (40.5%)
Associate degree	43 (12.4%)
Bachelor degree	73 (21.1%)
Postgraduate degree	22 (6.4%)
Marital status	
Married	176 (50.9%)
Divorced	33 (9.5%)
Separated	5 (1.4%)
Never married	132 (38.2%)
Household income (past year)	
< $24,999	40 (11.5%)
$25,000 to $34,999	26 (7.5%)
$35,000 to $49,999	52 (15.0%)
$50,000 to $74,999	68 (19.7%)
$75,000 to $99,999	62 (17.9%)
$100,000 to $199,999	77 (22.3%)
$200,000 or more	9 (2.6%)
Don't know	8 (2.3%)
Decline to answer	4 (1.2%)

TNP use

**Figure 2:**
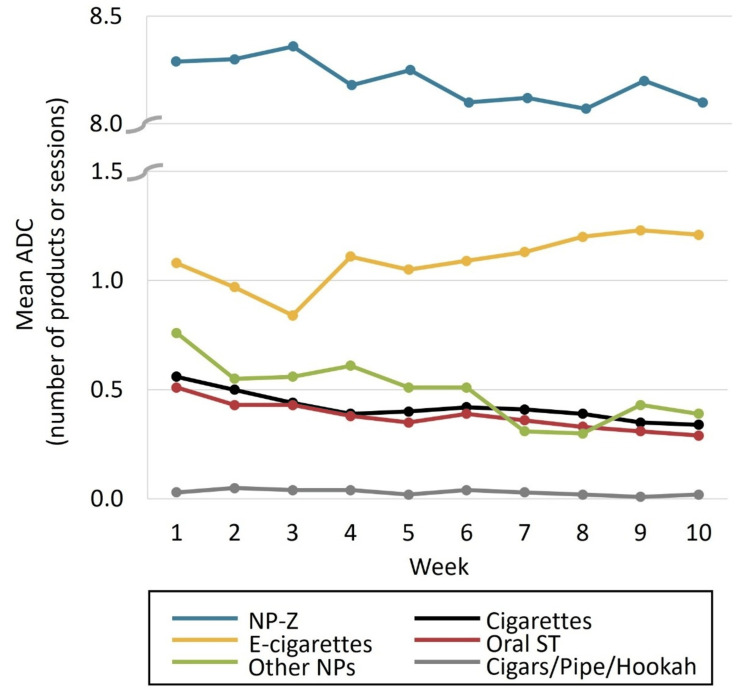
Mean average daily consumption (ADC) by tobacco and/or nicotine product type Full dataset shown in Table 2. The y-axis is broken to facilitate visualization of both ends of the scale. Oral smokeless tobacco (ST) includes moist snuff, chewing tobacco, and snus. ADC is the number of products consumed for cigarettes, cigars, nicotine pouches (NP), snus, moist snuff (dips), and chewing tobacco (pinches); number of sessions for e-cigarettes, pipe tobacco, and hookah/water pipe.

Average daily use: The mean ADC of TNPs over 10 weeks is depicted in Figure 2, with the full dataset in Table 2. The mean ADC for NP-Z was 8.29 pouches at week 1 and 8.10 pouches at week 10, corresponding to just over half a US canister per day. Although the participants had already been using NP-Z before the study, daily cigarette and oral ST use both trended down from week 1 to 10 (0.56 to 0.34 cigarettes and 0.51 to 0.29 products/dips/pinches, respectively).

**Table 2:** Mean ADC by TNP over the 10-week study period ^a^ Average daily reported TNP use was calculated based on the number of non-missing days for each week in the study period. Incremental and supplemental use was evaluated for NP-Z users based on the average daily reported use outcome. ^b^ Number of products consumed for cigarettes, cigars, NPs, snus, moist snuff (dips), and chewing tobacco (pinches); number of sessions for e-cigarettes, pipe tobacco, and hookah/water pipe. ^c^ Includes cigarillos and filtered cigars filled with tobacco. ADC, average daily consumption; NP, nicotine pouch; TNP, tobacco and/or nicotine product

Variables	Week									
Reported TNP use ^b^	1	2	3	4	5	6	7	8	9	10
	Mean ± SD^ b^									
NP-Z	8.29 ± 4.76	8.30 ± 4.68	8.36 ± 5.09	8.18 ± 5.08	8.25 ± 5.05	8.10 ± 5.20	8.12 ± 5.07	8.07 ± 5.37	8.20 ± 5.40	8.10 ± 5.38
Other NPs	0.76 ± 2.27	0.55 ± 1.93	0.56 ± 1.94	0.61 ± 2.10	0.51 ± 1.90	0.51 ± 2.09	0.31 ± 1.44	0.30 ± 1.28	0.43 ± 1.96	0.39 ± 1.78
Cigarettes	0.56 ± 2.34	0.50 ± 2.25	0.44 ± 2.13	0.39 ± 1.90	0.40 ± 2.05	0.42 ± 2.16	0.41 ± 2.05	0.39 ± 2.03	0.35 ± 1.87	0.34 ± 1.86
E-cigarettes	1.08 ± 5.80	0.97 ± 5.97	0.84 ± 5.08	1.11 ± 6.50	1.05 ± 6.47	1.09 ± 6.69	1.13 ± 6.98	1.20 ± 7.40	1.23 ± 7.88	1.21 ± 7.76
Moist snuff	0.43 ± 1.63	0.35 ± 1.37	0.34 ± 1.47	0.30 ± 1.30	0.28 ± 1.14	0.30 ± 1.35	0.29 ± 1.34	0.25 ± 1.27	0.27 ± 1.33	0.26 ± 1.41
Snus	0.07 ± 0.43	0.08 ± 0.51	0.09 ± 0.57	0.08 ± 0.49	0.07 ± 0.43	0.09 ± 0.55	0.07 ± 0.51	0.08 ± 0.59	0.04 ± 0.40	0.03 ± 0.34
Chewing tobacco	0.01 ± 0.13	0.00 ± 0.02	0.00 ± 0.08	0.00 ± 0.02	0.00 ± 0.02	0.00 ± 0.02	0.00 ± 0.04	0.00 ± 0.03	0.00 ± 0.00	0.00 ± 0.02
Cigars ^c^	0.02 ± 0.19	0.02 ± 0.20	0.02 ± 0.25	0.02 ± 0.26	0.01 ± 0.18	0.02 ± 0.17	0.01 ± 0.16	0.01 ± 0.10	0.01 ± 0.13	0.01 ± 0.07
Pipe tobacco	0.01 ± 0.09	0.02 ± 0.20	0.02 ± 0.23	0.02 ± 0.23	0.01 ± 0.21	0.02 ± 0.20	0.02 ± 0.26	0.01 ± 0.12	0.00 ± 0.07	0.01 ± 0.10
Hookah/water pipe	0.00 ± 0.04	0.01 ± 0.12	0.00 ± 0.02	0.00 ± 0.02	0.00 ± 0.02	0.00 ± 0.02	0.00 ± 0.01	0.00 ± 0.02	0.00 ± 0.02	0.00 ± 0.02

Weekly patterns of use: The proportions of participants using each TNP every day or some days (based on reported weekly use) are described in Table 3. The proportion of participants who smoked cigarettes at least once a week decreased from 15.9% (12.0-19.8%) at week 1 to 8.1% (5.2-11.0%) at week 10. The percentages of participants who used moist snuff at least once a week were 15.0% (11.2-18.8%) and 7.5% (4.7-10.3%) at weeks 1 and 10, respectively. Nearly one-quarter (24.0% (19.5-28.5%)) of participants who used NP-Z and other TNPs at baseline reported exclusive NP-Z use by the end of the study.

**Table 3:** Participants using each type of TNP some/every day based on reported weekly use over the 10-week study period ^a^ Includes cigarillos and filtered cigars filled with tobacco. CI, confidence interval; NA, not applicable; NP, nicotine pouch; TNP, tobacco and/or nicotine product

TNP	Week 1	Week 2	Week 3	Week 4	Week 5	Week 6	Week 7	Week 8	Week 9	Week 10
	n (%) (95% CI)									
NP-Z	343 (99.1%)	343 (98.6%)	338 (97.7%)	336 (97.1%)	334 (96.5%)	335 (96.8%)	332 (96.0%)	327 (94.5%)	323 (93.4%)	324 (93.6%)
	(98.2‒100.0%)	(97.3‒99.8%)	(96.1‒99.3%)	(95.3‒98.9%)	(94.6‒98.5%)	(95.0‒98.7%)	(93.9‒98.0%)	(92.1‒96.9%)	(90.7‒96.0%)	(91.1‒96.2%)
Other NPs	51 (14.7%)	34 (9.8%)	39 (11.3%)	41 (11.8%)	35 (10.1%)	31 (9.0%)	23 (6.6%)	23 (6.6%)	25 (7.2%)	23 (6.6%)
	(11.0‒18.5%)	(6.7‒13.0%)	(7.9‒14.6%)	(8.4‒15.3%)	(6.9‒13.3%)	(5.9‒12.0%)	(4.0‒9.3%)	(4.0‒9.3%)	(4.5‒10.0%)	(4.0‒9.3%)
Cigarettes	55 (15.9%)	49 (14.2%)	37 (10.7%)	32 (9.2%)	38 (11.0%)	32 (9.2%)	38 (11.0%)	33 (9.5%)	30 (8.7%)	28 (8.1%)
	(12.0‒19.8%)	(10.5‒17.9%)	(7.4‒14.0%)	(6.2‒12.3%)	(7.7‒14.3%)	(12.0‒19.8%)	(7.7‒14.3%)	(6.4‒12.6%)	(5.7‒11.7%)	(5.2‒11.0%)
E-cigarettes	36 (10.4%)	32 (9.2%)	30 (8.7%)	28 (8.1%)	30 (8.7%)	25 (7.2%)	30 (8.7%)	27 (7.8%)	24 (6.9%)	26 (7.5%)
	(7.2‒13.6%)	(6.2‒12.3%)	(5.7‒11.7%)	(5.2‒11.0%)	(5.7‒11.7%)	(4.5‒10.0%)	(5.7‒11.7%)	(5.0‒10.6%)	(4.2‒9.6%)	(4.7‒10.3%)
Moist snuff	52 (15.0%)	47 (13.6%)	37 (10.7%)	41 (11.8%)	38 (11.0%)	35 (10.1%)	26 (7.5%)	27 (7.8%)	28 (8.1%)	26 (7.5%)
	(11.2‒18.8%)	(10.0‒17.2%)	(7.4‒14.0%)	(8.4‒15.3%)	(7.7‒14.3%)	(6.9‒13.3%)	(4.7‒10.3%)	(5.0‒10.6%)	(5.2‒11.0%)	(4.7‒10.3%)
Snus	21 (6.1%)	14 (4.0%)	16 (4.6%)	16 (4.6%)	15 (4.3%)	20 (5.8%)	16 (4.6%)	12 (3.5%)	7 (2.0%)	9 (2.6%)
	(3.5‒8.6%)	(2.0‒6.1%)	(2.4‒6.8%)	(2.4‒6.8%)	(2.2‒6.5%)	(3.3‒8.3%)	(2.4‒6.8%)	(1.5‒5.4%)	(0.5‒3.5%)	(0.9‒4.3%)
Chewing tobacco	7 (2.0%)	2 (0.6%)	3 (0.9%)	2 (0.6%)	2 (0.6%)	2 (0.6%)	2 (0.6%)	2 (0.6%)	0 (0.0%)	3 (0.9%)
	(0.5‒3.5%)	(0.0‒1.4%)	(0.0‒1.8%)	(0.0‒1.4%)	(0.0‒1.4%)	(0.0‒1.4%)	(0.0‒1.4%)	(0.0‒1.4%)	NA	(0.0‒1.8%)
Cigars ^a^	11 (3.2%)	12 (3.5%)	10 (2.9%)	11 (3.2%)	11 (3.2%)	11 (3.2%)	4 (1.2%)	5 (1.4%)	7 (2.0%)	9 (2.6%)
	(1.3‒5.0%)	(1.5‒5.4%)	(1.1‒4.7%)	(1.3‒5.0%)	(1.3‒5.0%)	(1.3‒5.0%)	(0.0‒2.3%)	(0.2‒2.7%)	(0.5‒3.5%)	(0.9‒4.3%)
Pipe tobacco	5 (1.4%)	6 (1.7%)	4 (1.2%)	3 (0.9%)	5 (1.4%)	8 (2.3%)	3 (0.9%)	2 (0.6%)	1 (0.3%)	3 (0.9%)
	(0.2‒2.7%)	(0.4‒3.1%)	(0.0‒2.3%)	(0.0‒1.8%)	(0.2‒2.7%)	(0.7‒3.9%)	(0.0‒1.8%)	(0.0‒1.4%)	NA	(0.0‒1.8%)
Hookah/water pipe	5 (1.4%)	3 (0.9%)	3 (0.9%)	2 (0.6%)	2 (0.6%)	4 (1.2%)	2 (0.6%)	5 (1.4%)	1 (0.3%)	1 (0.3%)
	(0.2‒2.7%)	(0.0‒1.8%)	(0.0‒1.8%)	(0.0‒1.4%)	(0.0‒1.4%)	(0.0‒2.3%)	(0.0‒1.4%)	(0.2‒2.7%)	NA	NA

**Figure 3:**
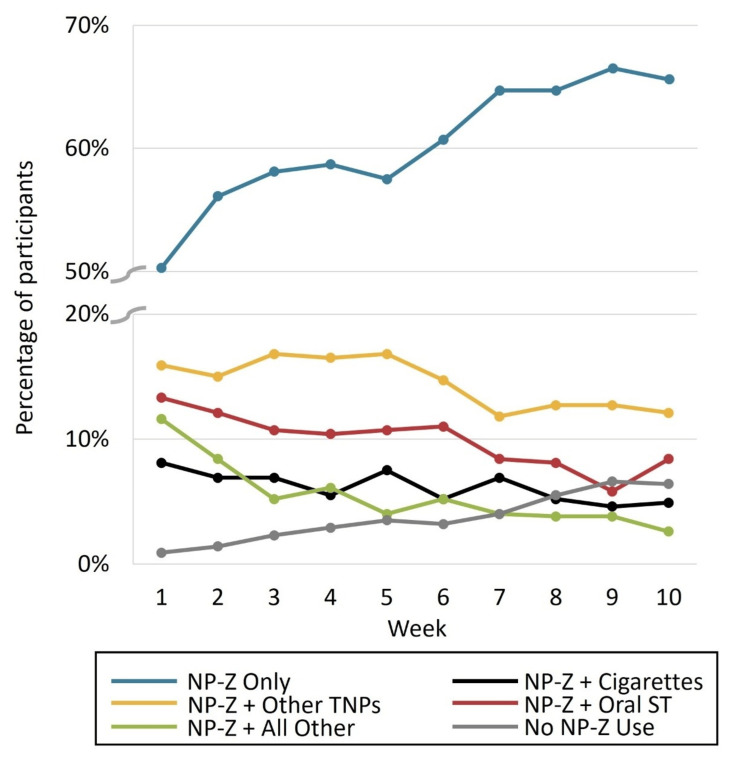
Single, dual, and poly TNP use Full dataset shown in Table 4. The y-axis is broken to facilitate visualization of both ends of the scale. Oral smokeless tobacco (ST) includes moist snuff, chewing tobacco, and snus; other tobacco and/or nicotine product (TNP) is all excluding cigarettes and oral ST; all other combinations include nicotine pouch (NP)-Z + cigarettes + snus, NP-Z + cigarettes + cigars, NP-Z + snus + cigars, etc.

Figure 3 shows an overview of the use of NP-Z alone or with other TNPs during the study; the full dataset is provided in Table 4. Over 10 weeks, the proportion of participants who exclusively used NP-Z increased from 50.3% to 65.6%. Corresponding decreases were shown for dual/poly use categories. Nearly half of the participants who reported cigarette use at baseline were not smoking in week 10 (8.1% (5.2-11.0%) to 4.9% (2.6-7.2%)).

**Table 4:** Single, dual, and poly TNP use over the 10-week study period ^a^ Moist snuff, chewing tobacco, and/or snus. ^b^ Not cigarettes or oral ST. ^c^ Combinations such as NP-Z + cigarettes + snus, NP-Z + cigarettes + cigars, NP-Z + snus + cigars, etc. CI, confidence interval; NP, nicotine pouch; ST, smokeless tobacco; TNP, tobacco and/or nicotine product

TNP	Week 1	Week 2	Week 3	Week 4	Week 5	Week 6	Week 7	Week 8	Week 9	Week 10
	n (%) (95% CI)									
TNP use (exclusive categories)										
NP-Z only	174 (50.3%)	194 (56.1%)	201 (58.1%)	203 (58.7%)	199 (57.5%)	210 (60.7%)	224 (64.7%)	224 (64.7%)	230 (66.5%)	227 (65.6%)
	(45.0‒55.6%)	(50.8‒61.3%)	(52.9‒63.3%)	(53.5‒63.9%)	(52.3‒62.7%)	(55.5‒65.9%)	(59.7‒69.8%)	(59.7‒69.8%)	(61.5‒71.5%)	(60.6‒70.6%)
NP-Z + cigarettes	28 (8.1%)	24 (6.9%)	24 (6.9%)	19 (5.5%)	26 (7.5%)	18 (5.2%)	24 (6.9%)	18 (5.2%)	16 (4.6%)	17 (4.9%)
	(5.2‒11.0%)	(4.2‒9.6%)	(4.2‒9.6%)	(3.1‒7.9%)	(4.7‒10.3%)	(2.9‒7.6%)	(4.2‒9.6%)	(2.9‒7.6%)	(2.4‒6.8%)	(2.6‒7.2%)
NP-Z + oral ST^ a^	46 (13.3%)	42 (12.1%)	37 (10.7%)	36 (10.4%)	37 (10.7%)	38 (11.0%)	29 (8.4%)	28 (8.1%)	20 (5.8%)	29 (8.4%)
	(9.7‒16.9%)	(8.7‒15.6%)	(7.4‒14.0%)	(7.2‒13.6%)	(7.4‒14.0%)	(7.7‒14.3%)	(5.4‒11.3%)	(5.2‒11.0%)	(3.3‒8.3%)	(5.4‒11.3%)
NP-Z + other TNP^ b^	55 (15.9%)	52 (15.0%)	58 (16.8%)	57 (16.5%)	58 (16.8%)	51 (14.7%)	41 (11.8%)	44 (12.7%)	44 (12.7%)	42 (12.1%)
	(12.0‒19.8%)	(11.2‒18.8%)	(12.8‒20.7%)	(12.5‒20.4%)	(12.8‒20.7%)	(11.0‒18.5%)	(8.4‒15.3%)	(9.2‒16.2%)	(9.2‒16.2%)	(8.7‒15.6%)
NP-Z + all other ^c^	40 (11.6%)	29 (8.4%)	18 (5.2%)	21 (6.1%)	14 (4.0%)	18 (5.2%)	14 (4.0%)	13 (3.8%)	13 (3.8%)	9 (2.6%)
	(8.2‒14.9%)	(5.4‒11.3%)	(2.9‒7.6%)	(3.5‒8.6%)	(2.0‒6.1%)	(2.9‒7.6%)	(2.0‒6.1%)	(1.7‒5.8%)	(1.7‒5.8%)	(0.9‒4.3%)
No NP-Z use	3 (0.9%)	5 (1.4%)	8 (2.3%)	10 (2.9%)	12 (3.5%)	11 (3.2%)	14 (4.0%)	19 (5.5%)	23 (6.6%)	22 (6.4%)
	(0.0‒1.8%)	(0.2‒2.7%)	(0.7‒3.9%)	(1.1‒4.7%)	(1.5‒5.4%)	(1.3‒5.0%)	(2.0‒6.1%)	(3.1‒7.9%)	(4.0‒9.3%)	(3.8‒8.9%)

Intention to quit: MTSS scores for each type of TNP were collected biweekly (Table 5). There were seven response options ranging from one to seven (lowest and highest motivation to stop, respectively). Across the TNP type, the MTSS scores remained fairly consistent during the 10-week study. At week 10, participants expressed the highest desire to quit cigarettes (5.38), followed by oral ST (5.27 for snus, 4.77 for moist snuff), and e-cigarettes (3.70). The mean MTSS score for NP-Z was 2.62, which falls between “I think I should stop but don’t really want to” and “I want to stop but haven’t thought about when.”

**Table 5:** MTSS scores (mean ± SD) a for intention of participants to quit TNP use (by type) during the 10-week study period ^a^ Response options: (1) “I don’t want to stop,” (2) “I think I should stop but don’t really want to,” (3) “I want to stop but haven’t thought about when,” (4) “I really want to stop but I don’t know when I will,” (5) “I want to stop and hope to soon,” (6) “I really want to stop and intend to in the next 3 months,” and (7) “I really want to stop smoking and intend to in the next month.” ^b^ Number of non-missing responses for the MTSS. ^c^ Respondents who used NP-Z in the past 2 weeks did not answer about their intention to quit other NPs. ^d^ Includes cigarillos and filtered cigars filled with tobacco. MTSS, Motivation to Stop Scale; NP, nicotine pouch; TNP, tobacco and/or nicotine product

Intention to quit	Week 2		Week 4		Week 6		Week 8		Week 10	
	n^ b^	Score	n^ b^	Score	n^ b^	Score	n ^b^	Score	n^ b^	Score
NP-Z	308	2.23 ± 1.44	292	2.42 ± 1.58	294	2.43 ± 1.64	300	2.62 ± 1.75	320	2.62 ± 1.70
Other NPs^ c^	0		0		0		0		0	
Cigarettes	53	5.26 ± 1.69	30	4.80 ± 1.97	35	5.86 ± 1.73	38	5.42 ± 1.97	34	5.38 ± 1.86
E-cigarettes	32	2.88 ± 1.70	29	3.48 ± 2.20	25	3.28 ± 2.05	29	3.38 ± 2.18	27	3.70 ± 2.11
Moist snuff	51	4.94 ± 2.06	42	4.60 ± 1.99	44	5.25 ± 1.89	30	4.33 ± 2.14	30	4.77 ± 2.34
Snus	24	3.58 ± 2.45	19	4.05 ± 2.46	19	5.16 ± 2.29	17	3.47 ± 2.45	11	5.27 ± 2.28
Chewing tobacco	7	5.29 ± 2.06	4	4.75 ± 2.63	3	3.00 ± 2.65	2	5.00 ± 0.00	2	3.50 ± 3.54
Cigars ^d^	20	3.65 ± 2.37	12	4.25 ± 2.49	12	3.50 ± 2.54	5	2.60 ± 2.51	11	2.45 ± 2.07
Pipe tobacco	7	3.00 ± 2.52	4	4.25 ± 2.63	6	4.83 ± 2.48	3	4.67 ± 2.52	3	3.67 ± 2.89
Hookah/water pipe	7	3.00 ± 2.08	3	2.33 ± 1.53	4	3.50 ± 2.52	3	5.00 ± 3.46	1	1.00

At the end of the study, 11 (3.2% (1.3-5.0%)) participants had quit all TNPs. Eighty-three (24.0% (19.5-28.5%)) participants who had reported dual use in week 1 reported complete replacement of NP-Z in place of other TNPs in weeks 9 and 10.

Reasons for NP-Z use: Participants selected their reasons for NP-Z use in the biweekly survey (Table 6). Although some shifts occurred over 10 weeks, the most commonly selected reasons were similar at the beginning and end of the study. Among participants who smoked at week 10, 84.0% selected “Help reduce cigarette smoking,” and 60.0% chose “Help me quit smoking.”

**Table 6:** Reasons for NP-Z use ^a^ Number of respondents who completed the survey and were eligible to select the answer. NP, nicotine pouch; TNP, tobacco and/or nicotine product

Reason	Week 2		Week 4		Week 6		Week 8		Week 10	
	n^ a^	Value	n^ a^	Value	n^ a^	Value	n^ a^	Value	n^ a^	Value
To help me reduce my cigarette smoking	49	30 (61.2%)	25	16 (64.0%)	30	24 (80.0%)	28	20 (71.4%)	25	21 (84.0%)
To help me quit smoking cigarettes	49	31 (63.3%)	25	15 (60.0%)	30	24 (80.0%)	28	19 (67.9%)	25	15 (60.0%)
To help me reduce my use of tobacco products other than cigarettes	307	87 (28.3%)	291	95 (32.6%)	295	88 (29.8%)	297	100 (33.7%)	320	93 (29.1%)
To help me quit using tobacco products other than cigarettes	308	115 (37.3%)	292	114 (39.0%)	295	108 (36.6%)	299	108 (36.1%)	320	111 (34.7%)
To use in environments where other TNPs are not considered appropriate (e.g., church)	307	153 (49.8%)	290	149 (51.4%)	294	156 (53.1%)	298	163 (54.7%)	320	202 (63.1%)
To use in environments where other TNPs are not allowed (e.g., airplane)	307	148 (48.2%)	291	149 (51.2%)	295	163 (55.3%)	298	163 (54.7%)	320	187 (58.4%)
Less harmful to my health than cigarettes	309	142 (46.0%)	292	149 (51.0%)	297	153 (51.5%)	300	144 (48.0%)	320	175 (54.7%)
Less harmful to my health than other tobacco products, excluding cigarettes	310	172 (55.5%)	293	152 (51.9%)	298	156 (52.3%)	298	156 (52.3%)	320	156 (48.8%)
To avoid spitting as required with other products	307	143 (46.6%)	291	131 (45.0%)	294	133 (45.2%)	298	156 (52.3%)	320	163 (50.9%)
To add variety to the products I use	305	11 (3.6%)	289	18 (6.2%)	293	13 (4.4%)	296	22 (7.4%)	320	17 (5.3%)
Comes in flavors I like	308	143 (46.4%)	293	147 (50.2%)	296	147 (49.7%)	298	143 (48.0%)	320	165 (51.6%)
Does not cause me to smell like smoke/tobacco	306	149 (48.7%)	293	145 (49.5%)	294	144 (49.0%)	297	155 (52.2%)	320	168 (52.5%)
Comes in two different levels of nicotine strength	307	40 (13.0%)	291	40 (13.7%)	294	48 (16.3%)	297	52 (17.5%)	320	58 (18.1%)
Less harmful for those around me than cigarettes	306	101 (33.0%)	291	114 (39.2%)	294	116 (39.5%)	298	120 (40.3%)	320	126 (39.4%)
More acceptable to non-tobacco users	305	122 (40.0%)	291	113 (38.8%)	294	136 (46.3%)	297	136 (45.8%)	320	148 (46.3%)
No one can tell when I am using it	307	153 (49.8%)	291	169 (58.1%)	295	182 (61.7%)	298	196 (65.8%)	320	209 (65.3%)
I was just curious to see what it was like	305	20 (6.6%)	289	14 (4.8%)	293	13 (4.4%)	296	18 (6.1%)	320	21 (6.6%)
Ease of use	307	179 (58.3%)	292	176 (60.3%)	295	184 (62.4%)	298	189 (63.4%)	320	217 (67.8%)
Recommended by person who works in the store where I buy my TNPs	305	13 (4.3%)	289	10 (3.5%)	293	11 (3.8%)	296	11 (3.7%)	320	14 (4.4%)
None of the above	305	4 (1.3%)	289	3 (1.0%)	293	4 (1.4%)	296	3 (1.0%)	320	2 (0.6%)
Don't know	305	1 (0.3%)	289	1 (0.3%)	293	2 (0.7%)	296	0 (0%)	320	0 (0%)
Decline to answer	305	0 (0%)	289	0 (0%)	293	0 (0%)	296	0 (0%)	320	0 (0%)

Adverse events: Two non-serious AEs (cold symptoms) were reported by two participants, with no follow-up required.

## Discussion

The present observational study - conducted in 2017 and 2018 when NPs were new to the US market - examined patterns of all TNP use and intention to quit TNP over a 10-week period in participants who were early NP-Z adopters. The results show changes in TNP use behaviors among a sample of early NP-Z adopters. Although they had already started using the product before the study, the results suggest that their use patterns were still in a period of transition. Exclusive NP-Z increased over the 10 weeks, while concurrent NP-Z and cigarette use meaningfully decreased. Further, participants who smoked and used NP-Z maintained high motivation to quit smoking. To contextualize these findings, it is important to consider how national patterns of TNP use have evolved since 2018. Doing so helps assess whether the behaviors observed among early adopters reflect broader population-level trends and informs the potential public health impact of NPs as they gain market share.

The US TNP landscape has changed dramatically since the time of this study, with shifts from cigarette smoking to the use of newer TNPs, but little is known about NP patterns of use. PATH data from December 2016 to January 2018 (Wave 4) indicate that 20.9%, 4.9%, and 3.1% of US adults ≥25 years old or older had smoked cigarettes, used e-cigarettes, or used oral ST in the past 30 days, respectively [16-18]. The National Health Interview Survey (NHIS) data collected from 2017 to 2023 showed that ~6.8 million fewer US adults exclusively smoked cigarettes, but ~7.2 million more adults exclusively used e-cigarettes [19]. Unfortunately, these nationally representative surveys have only recently included questions on NP use. The National Youth Tobacco Survey was the first to ask about NPs in 2021, and in that year, 0.8% of middle/high school-age students reported past 30-day use (compared to 7.6% and 1.5% for e-cigarettes and cigarettes, respectively) [20]. The PATH study and the NHIS initially included questions about NP use in 2022 (Wave 7) and 2024, respectively.

At the individual level, a handful of smaller surveys have focused on TNP users. Sparrock et al. assessed NP awareness, use, susceptibility, and beliefs among 1,583 US adult current tobacco users (age ≥21) who participated in the COVID-19 and Commercial Tobacco Use Study in early 2021 and found ever and current use prevalence rates of 16.4% and 3.0%, respectively [21]. Current use was significantly more common among those who currently used cigarettes, e-cigarettes, or oral ST products. A cross-sectional analysis of Wave 3 (February-June 2020) of the International Tobacco Control Four Country Smoking and Vaping Survey included 2,507 US participants and reported 3.0% and 0.9% ever and current NP use [22]. Data from the 2022 Tobacco Use Supplement of the Current Population found that 2.9% and 0.4% of US adults surveyed had ever or currently used NPs, respectively [23]. An annual tobacco and nicotine use surveillance conducted among military personnel in 2022-23 found that NP use was 10-fold higher than estimated for the general population [24]. In all three studies, most respondents were male, young, White, and tobacco users. This aligns with the sociodemographic profile of the participants in the present study. A 2024 review reached similar conclusions but noted that studies focusing on the demographic characteristics of NP users had heterogeneous samples and focused on adult current/former TNP users [25]. Future data from the PATH and NHIS will provide more insight into whether NPs are replacing more harmful sources of nicotine at the population level. No nationally representative survey asks nuanced questions about NP use. Granular data are essential for understanding patterns of consumption that can inform harm reduction strategies. In the present study, NP-Z users reported a downward trend in cigarette and moist snuff use during the 10-week study period, even though they had used NP-Z for a median of 5-6 months. This is consistent with the reasonably high MTSS scores recorded among NP-Z users for intention to quit cigarettes, moist snuff, and/or snus during all 10 weeks. Similar results were reported in two six-week actual use studies that examined how use behavior changed when current smokers and ST users who were not planning to quit were offered NPs to use alongside their usual TNPs [26,27]. Becker et al. found that 27% of smokers and 71% of ST users reported that they had stopped using their usual product and only used NPs. Among those who continued dual use, 39% of smokers and 14% of ST users had reduced their usual product consumption by ≥50% [26]. Campbell et al. noted that 82% of participants had decreased average daily cigarette consumption, while 16% had reduced their cigarette consumption by ≥50% [27]. The findings from both studies suggested that NPs may have utility as a tobacco harm reduction tool; however, it was unclear whether reductions would be sustained after NPs were not freely provided. The real-world data described here suggest that NPs may be used to replace more harmful TNPs.

There is also a lack of information on why US adults use NPs. A cross-sectional survey to characterize NP use motives asked 118 respondents to select a primary motivation for NP use from a list of 12 options [28]. The top choice - selected by almost a third (31%) - was “It comes in flavors I like/liked.” Multiple options could be selected in the present study, and while “Comes in flavors I like” was selected by 51.6% of participants, it was not among the five most common answers. Instead, the main reasons selected by participants in the present study were related to reducing/quitting cigarette smoking and ease of use.

Strengths and limitations

The study provides the earliest data on detailed NP-Z patterns of use at a time when the product category was relatively unknown in the US. However, the results should be considered in the context of several limitations. First, only descriptive analyses are presented. Future study designs should include inferential statistical tests to formally evaluate differences between groups or over time. Second, the findings are not representative of the whole US population. They are only generalizable to US adults who were early adopters of NP-Z in late 2017/early 2018 and were residing in the 11 states where NP-Z was sold at the time. As a result, the study population is largely white and male. Third, the participants may have been established in their NP-Z use, leading to an underestimation of baseline cigarette use relative to those who have just started using NP-Z. Fourth, as nearly half of the participants failed to complete the 10-week study, selection bias may have overestimated the stability of NP-Z use and reduction in the use of other TNPs. Finally, the results were dependent on self-report and therefore subject to recall bias; however, any recall bias is likely to be constant across time points, and daily e-diary recordings were used to help mitigate this issue.

## Conclusions

The findings of this real-world study indicate that traditional TNP users are willing to buy, try, and use NP-Z, including as a replacement for other TNPs. Most participants were not smoking cigarettes at the time of the survey, but those who were expressed a high intention to quit smoking, and nearly half did at the end of the 10-week observation period. Cigarette smoking and oral ST use trended downwards, even though the participants had been using NP-Z for several months. Collectively, these results suggest that NP-Z can serve as a harm reduction option for people who smoke or use oral ST and would otherwise continue using their respective TNPs. Continued post-market surveillance is important to understand how adults who use TNPs shift their TNP use towards less harmful products. Longitudinal experimental studies are needed to confirm whether these products can effectively help adults switch from smoking cigarettes.

## Figures and Tables

**Figure 4:**
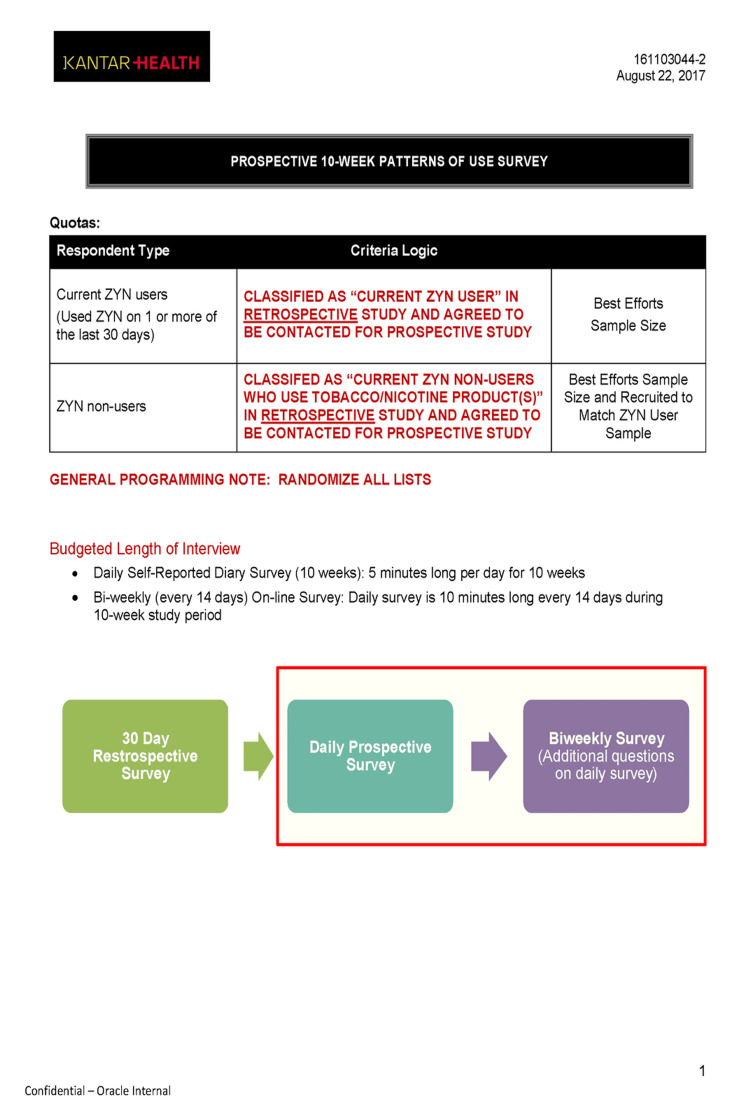
Prospective 10-week patterns of the use survey (page 1)

**Figure 5:**
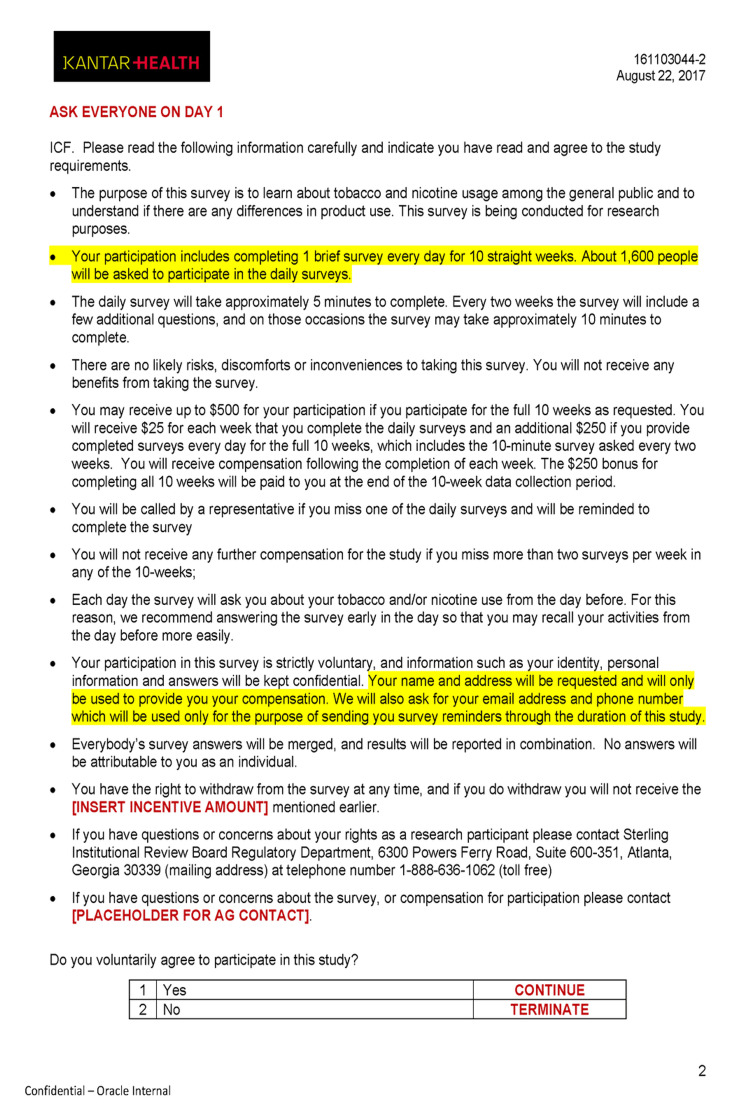
Prospective 10-week patterns of use survey (page 2)

**Figure 6:**
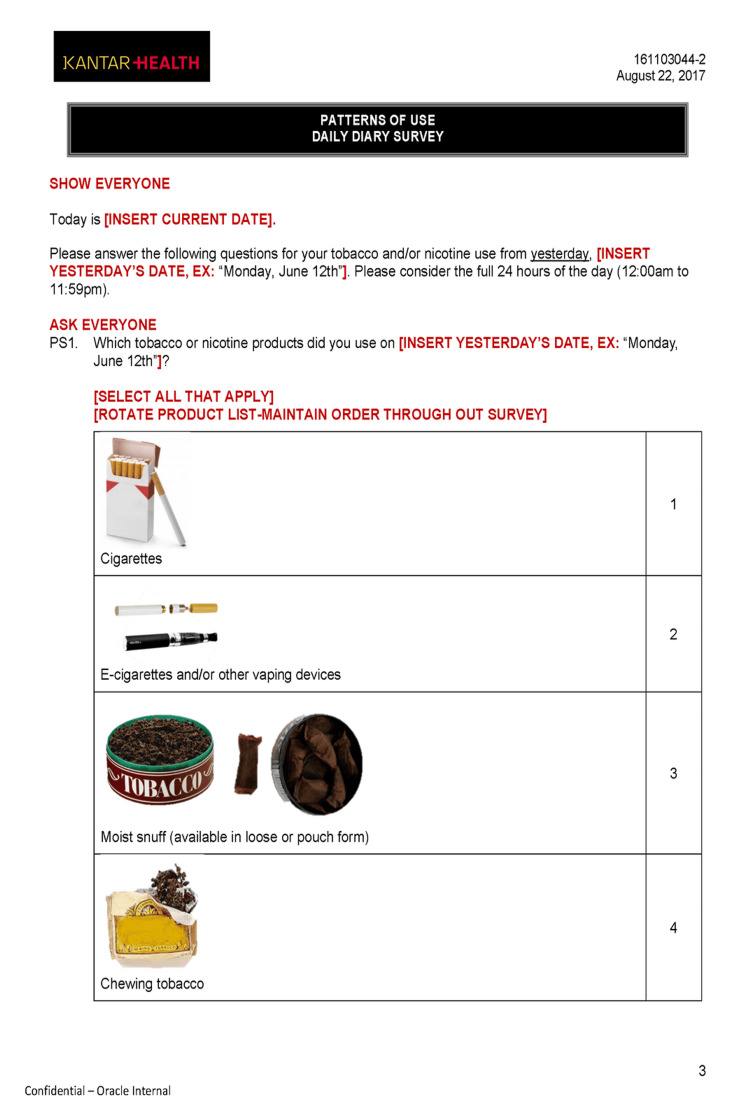
Prospective 10-week patterns of use survey (page 3)

**Figure 7:**
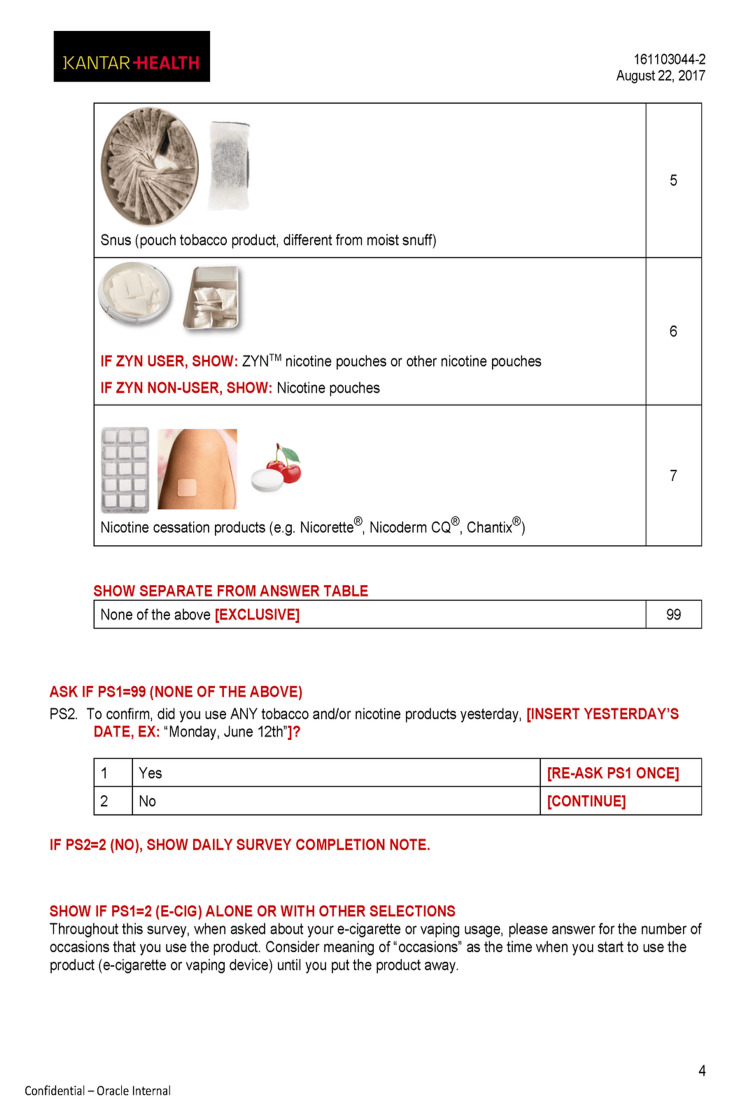
Prospective 10-week patterns of use survey (page 4)

**Figure 8:**
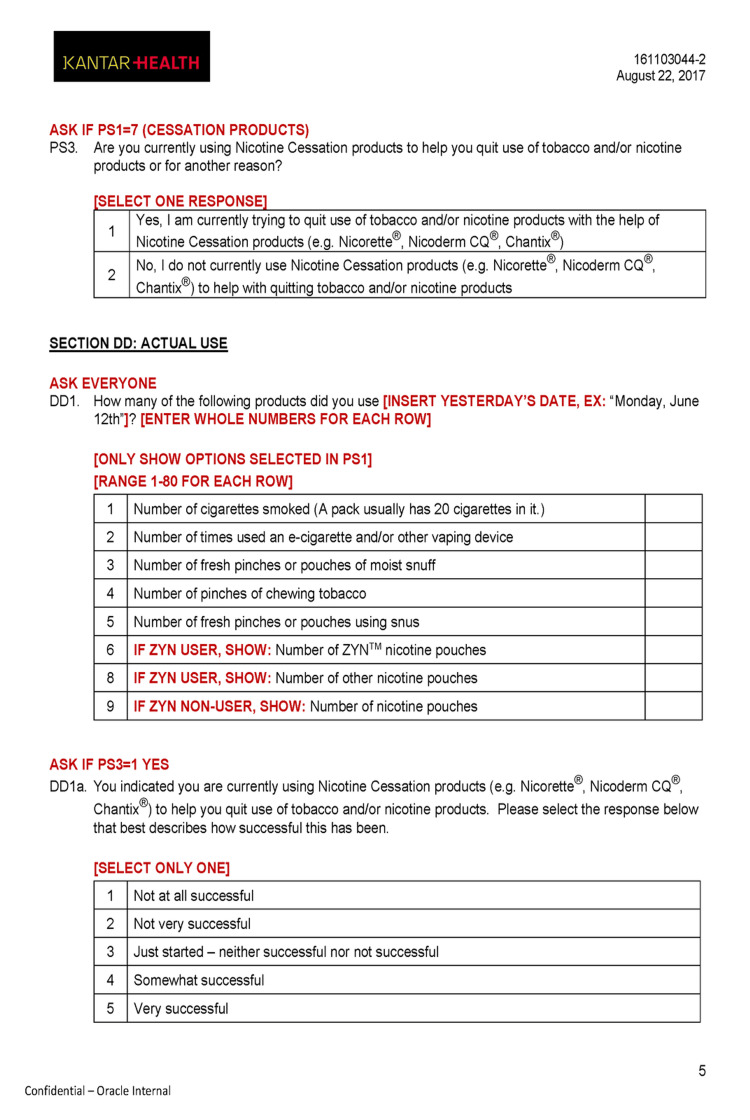
Prospective 10-week patterns of use survey (page 5)

**Figure 9:**
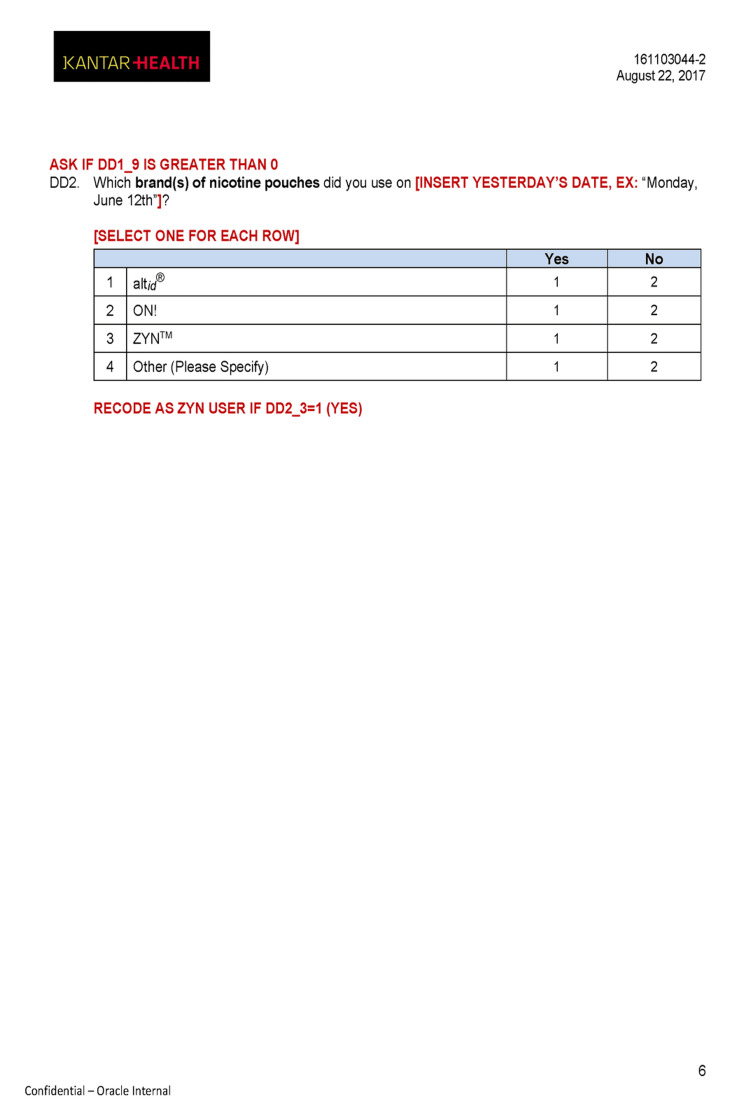
Prospective 10-week patterns of use survey (page 6)

**Figure 10:**
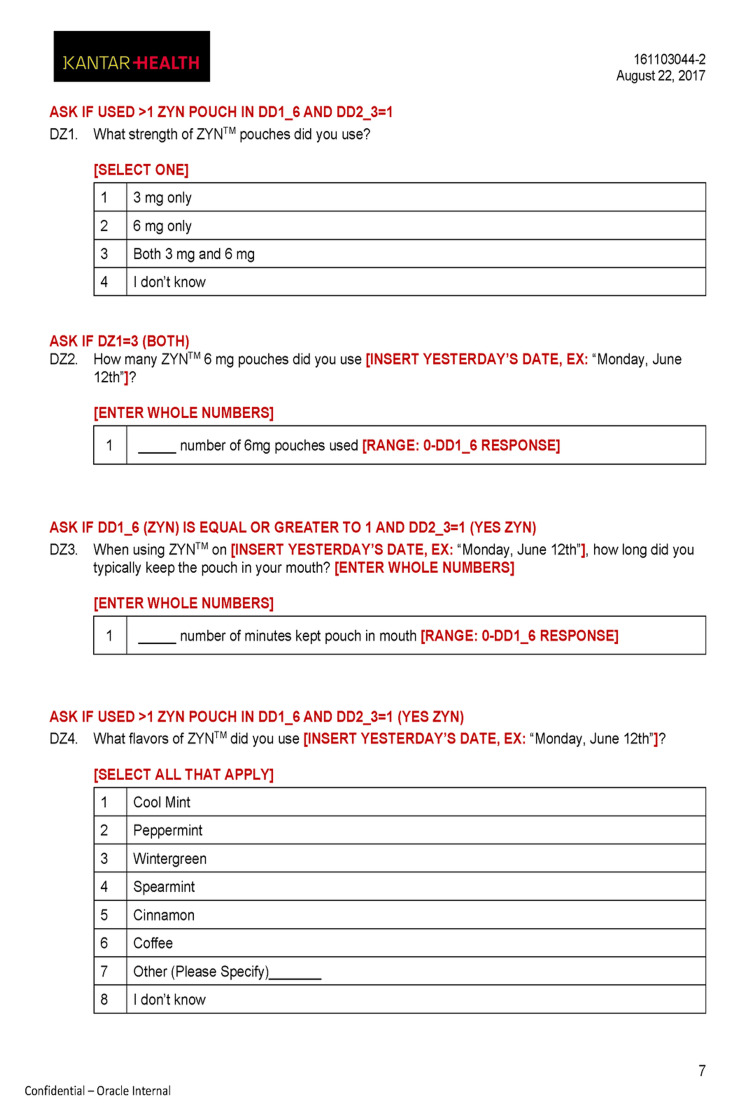
Prospective 10-week patterns of use survey (page 7)

**Figure 11:**
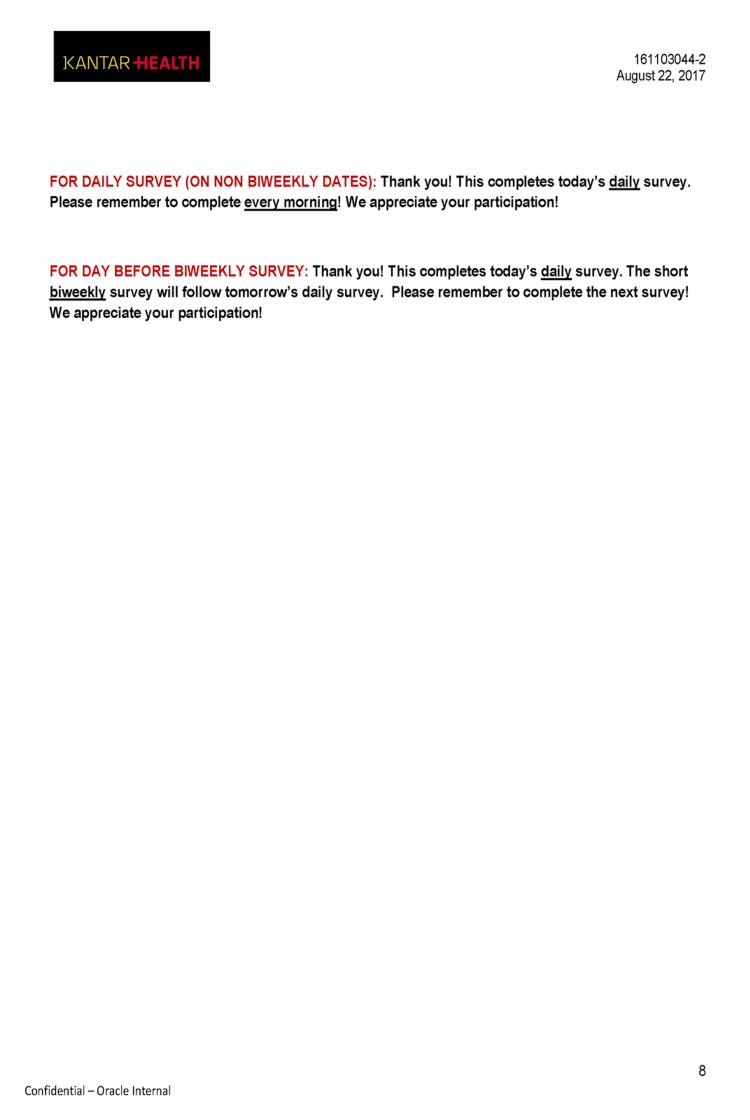
Prospective 10-week patterns of use survey (page 8)

**Figure 12:**
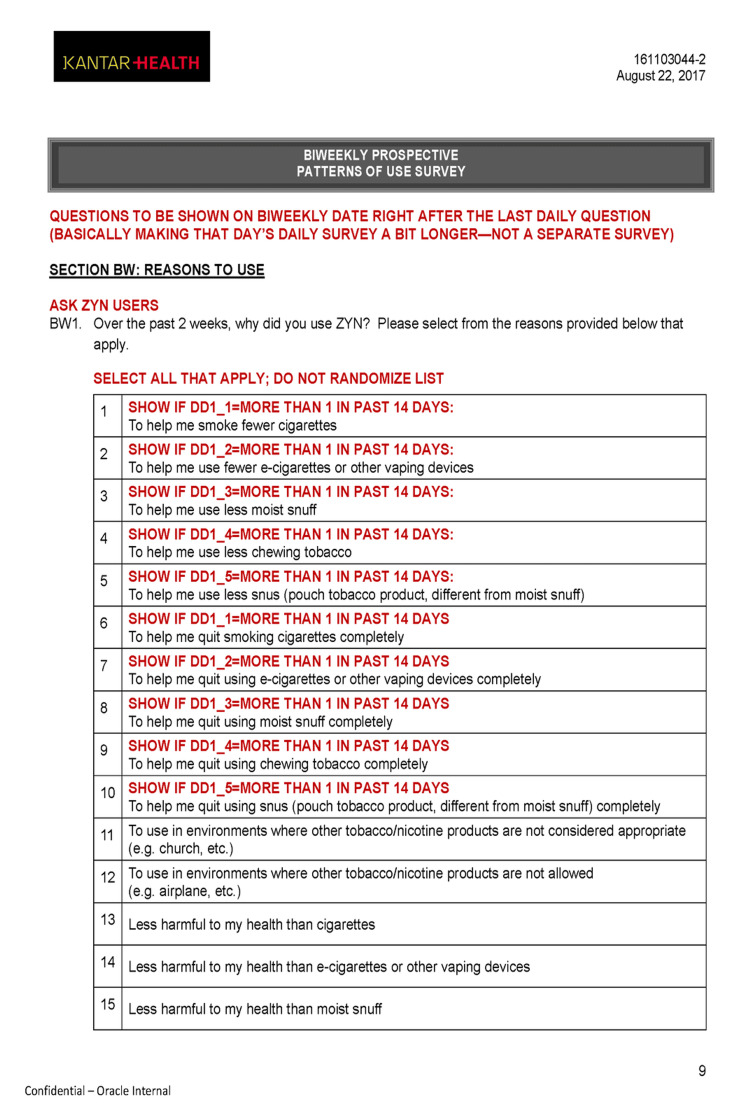
Prospective 10-week patterns of use survey (page 9)

**Figure 13:**
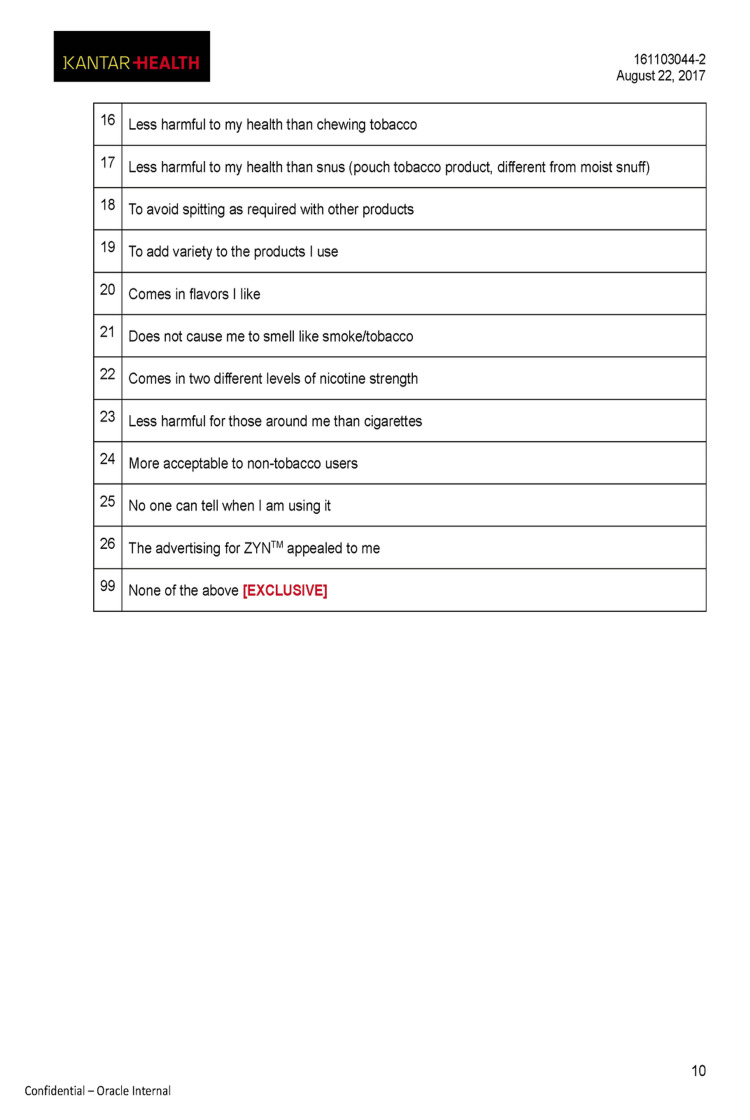
Prospective 10-week patterns of use survey (page 10)

**Figure 14:**
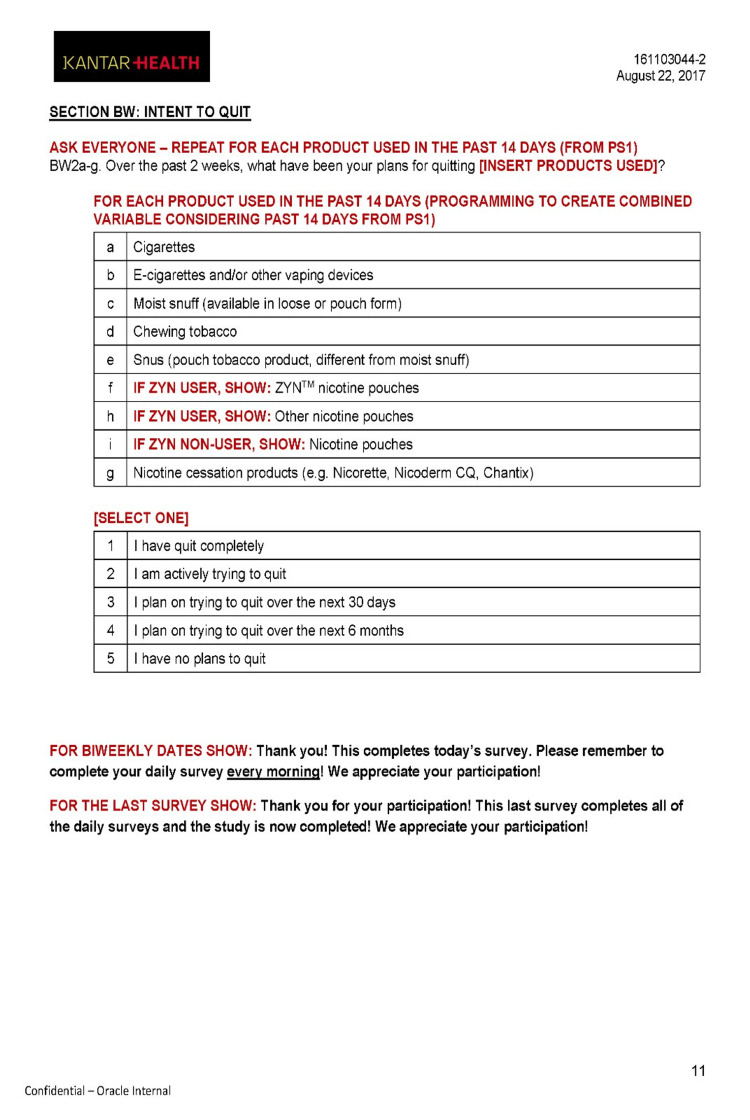
Prospective 10-week patterns of use survey (page 11)

## Appendices

Supplementary materials

Figures 4-14 present the survey form.
